# Some Accessions of Amazonian Wild Rice (*Oryza glumaepatula*) Constitutively Form a Barrier to Radial Oxygen Loss along Adventitious Roots under Aerated Conditions

**DOI:** 10.3390/plants9070880

**Published:** 2020-07-13

**Authors:** Masato Ejiri, Yuto Sawazaki, Katsuhiro Shiono

**Affiliations:** Graduate School of Bioscience and Biotechnology, Fukui Prefectural University, 4-1-1 Matsuoka-Kenjojima, Eiheiji, Fukui 910-1195, Japan; s2093001@g.fpu.ac.jp (M.E.); s2073004@g.fpu.ac.jp (Y.S.)

**Keywords:** apoplastic barrier, barrier to radial oxygen loss (ROL), lignin, *Oryza glumaepatula*, *O. rufipogon*, rice (*O. sativa*), suberin, wild rice

## Abstract

A barrier to radial oxygen loss (ROL), which reduces the loss of oxygen transported via the aerenchyma to the root tips, enables the roots of wetland plants to grow into anoxic/hypoxic waterlogged soil. However, little is known about its genetic regulation. Quantitative trait loci (QTLs) mapping can help to understand the factors that regulate barrier formation. Rice (*Oryza sativa*) *inducibly* forms an ROL barrier under stagnant conditions, while a few wetland plants *constitutively* form one under aerated conditions. Here, we evaluated the formation of a constitutive ROL barrier in a total of four accessions from two wild rice species. Three of the accessions were wetland accessions of *O. glumaepatula*, and the fourth was a non-wetland species of *O. rufipogon*. These species have an AA type genome, which allows them to be crossed with cultivated rice. The three *O. glumaepatula* accessions (W2165, W2149, and W1183) formed an ROL barrier under aerated conditions. The *O. rufipogon* accession (W1962) did not form a constitutive ROL barrier, but it formed an inducible ROL barrier under stagnant conditions. The three *O. glumaepatula* accessions should be useful for QTL mapping to understand how a constitutive ROL barrier forms. The constitutive barrier of W2165 was closely associated with suberization and resistance to penetration by an apoplastic tracer (periodic acid) at the exodermis but did not include lignin at the sclerenchyma.

## 1. Introduction

Under waterlogged conditions, plants can suffer from hypoxia or anoxia because the ability of oxygen to diffuse through the water to the soil is extremely low [1]. Other problems associated with waterlogging are the accumulation of phytotoxic compounds in the soil and a decline in the availability of some nutrients [2]. The roots of wetland plants contain a large volume of aerenchyma, which provides a low-resistance pathway for the diffusion of oxygen from the shoot to the root [3,4,5]. Some wetland species also form a barrier to radial oxygen loss (ROL) [2,6,7,8]. The ROL barrier forms at the basal part of roots and reduces the loss of oxygen transported via the aerenchyma to the root tips. In roots with an ROL barrier, oxygen in the root tips and short lateral roots can be maintained at a higher level to allow root elongation into hypoxic/anoxic soil [5,6]. An ROL barrier in roots is a key feature contributing to long-distance oxygen transport and waterlogging tolerance in wetland species.

Lignin and suberin act as an apoplastic diffusion barrier in the root. Lignin is a complex of polyphenolic polymers [9], which are the main components of Casparian strips [10]. Suberin is a hydrophobic macromolecule built from long-chain fatty acids, glycerol, and aromatic polymers [11], and is the main component of suberin lamellae [12]. An ROL barrier is thought to be formed by deposits of suberin in the hypodermis/exodermis but not by deposits of lignin [6,13]. Suberin deposits in the apoplast (the outer cellular space) prevent movement of ions and mycorrhizal fungi through the apoplast and thus act as an apoplastic barrier [14,15,16]. At the basal part, the ROL barrier, in addition to restricting oxygen loss, also could reduce the entry of soil phytotoxins (e.g., Fe^2+^) into roots in waterlogged soils [6,17]. Another possible benefit of the ROL barrier is that it would allow oxygen to reach the root tip. Because the root tip does not have an ROL barrier, the ROL at the root tip could detoxify toxic reduced substances in the waterlogged soil [2].

Some wetland plants, including cultivated rice (*Oryza sativa*), form an inducible ROL barrier under waterlogged soil or stagnant conditions, while they remain leaky to oxygen under well-drained or aerated conditions [2,4,18]. Some soil phytotoxins produced by anaerobic bacteria in waterlogged soils (e.g., Fe^2+^, NH_4_, sulfide, and/or carboxylic acids) seem to act as environmental triggers to induce ROL barrier formation [5,19,20,21], although the signaling pathway to induce the ROL barrier is not known [6]. On the other hand, a few wetland plants (e.g., wild *Echinochloa* species) form a constitutive ROL barrier, i.e., even in the absence of waterlogging [13,22,23]. 

The molecular mechanisms that control whether a plant forms an ROL barrier inducibly or constitutively are not understood. One approach to this problem is to map the quantitative trait loci (QTLs) that control constitutive ROL barrier formation. To do this in rice, it is necessary to have a rice accession that constitutively forms an ROL barrier. However, none of 14 rice cultivars examined for a constitutive ROL barrier were found to have one [24,25,26]. So far, 24 wild rice species have been identified in tropical regions [27]. Seven of them, including *O. glumaepatula* and *O. rufipogon*, have an AA-genome [27], the same as the genome of cultivated rice (*O. sativa*), which would allow cross-breeding. We thus examined three accessions of *O. glumaepatula* and one accession of *O. rufipogon* to see if they constitutively form an ROL barrier and aerenchyma. In one of the accessions that formed a constitutive ROL barrier, we evaluated its chemical composition and ability to act as an apoplastic barrier to better characterize its properties.

## 2. Results

### 2.1. Discovery of Wild Rice Accessions That Constitutively Form a Radial Oxygen Loss (ROL) Barrier

**Figure 1:**
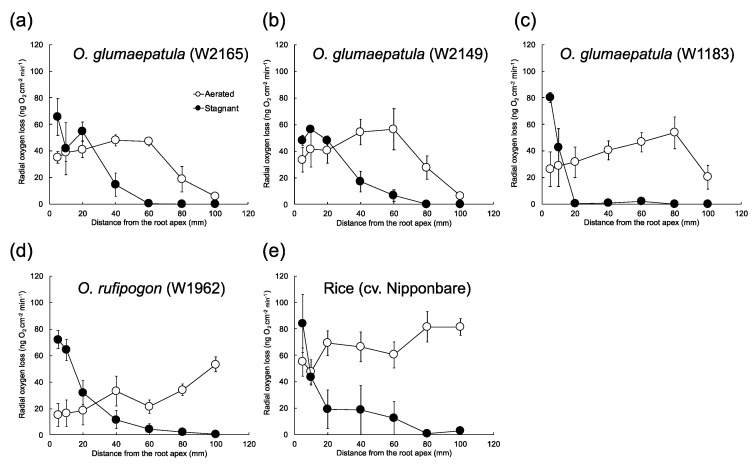
Rates of radial oxygen loss (ROL) along adventitious roots in accessions of *O. glumaepatula*, *O. rufipogon*, and rice (cv. Nipponbare) under aerated or stagnant conditions. W2165 (**a**), W2149 (**b**), and W1183 (**c**) of *O. glumaepatula* came from wetlands. W1962 (**d**) of *O. rufipogon* came from habitats other than wetlands. For a control that forms an inducible ROL barrier, cv. Nipponbare (**e**) was used. ROL along adventitious roots (115–120 mm length) was measured by Pt cylindrical electrode. Plants were grown in aerated nutrient solution for eight days and then transferred to deoxygenated stagnant 0.1% agar solution or continued aerated solution for 13–15 days. Mean ± standard error (SE). *n* = 3 or 4.

To determine whether the four wild rice accessions formed an ROL barrier under aerated conditions, oxygen leakage was measured along adventitious roots (about 120 mm long) in three wetland accessions of *O. glumaepatula* (W2165, W2149, and W1183) and one non-wetland accession of *O. rufipogon* (W1962) (Figure 1). Under aerated conditions, all three *O. glumaepatula* accessions (W2165, W2149 and W1183) had an oxygen loss of about 30 ng cm^−2^ min^−1^ in the apical 5 mm and much less oxygen loss (≤20 ng cm^−2^ min^−1^) in the basal region (Figure 1a–c, open circles), which are the characteristics of an ROL barrier. In the accessions of *O. rufipogon* (W1962) under aerated conditions, the oxygen flux from the basal part remained high (≥50 ng cm^−2^ min^−1^ at 100 mm from the apex) (Figure 1d, open circles), indicating that they did not form a constitutive ROL barrier.

### 2.2. Assessment of Inducible ROL Barrier Formation

When W2165, W2149, and W1183 of *O. glumaepatula* were grown under stagnant conditions, the barrier became stronger from the basal part to the middle part of the roots (Figure 1a–c, closed circles). The rates of ROL at the basal parts were close to zero, and oxygen leakage at 40 mm from the root apex was even lower (40 mm: under 20 ng cm^−2^ min^−1^). When W1962 of *O. rufipogon* was grown under stagnant conditions, the oxygen flux at the basal regions (40–100 mm from the root apex) was low (1–12 ng cm^−2^ min^−1^) (Figure 1d, closed circles), indicating it had a tight ROL barrier. When cv. Nipponbare was grown under stagnant conditions, the oxygen flux at the basal to middle regions (20–100 mm from the root apex) was low (1–19 ng cm^−2^ min^−1^) (Figure 1e, closed circles), indicating it had a tight barrier to ROL. In W2165 under aerated conditions, the rates of ROL at the basal part were comparable to those of cv. Nipponbare roots under stagnant conditions, although the region with a strong barrier in W2165 was smaller than the region with an inducible ROL barrier in cv. Nipponbare under stagnant conditions.

### 2.3. Assessment of Aerenchyma Formation

Under aerated conditions, all wild rice accessions of *O. glumaepatula* and *O. rufipogon* had well-developed aerenchyma [21 to 48% of the cortex cross-section at the basal part (100 mm from the root apex) (Table 1)]. These values were as high as the percentage in cv. Nipponbare, which indicates that these accessions form aerenchyma constitutively. All wild rice accessions formed aerenchyma constitutively. However, stagnant conditions induced additional increases in aerenchyma of almost 50% in three of the four wild accessions (W2149, W1183, and W1962) (Table 1). All wild rice accessions formed aerenchyma constitutively and induced aerenchyma by stagnant treatment, but not superior to cultivated rice cv. Nipponbare. 

### 2.4. Suberin and Lignin Accumulation in W2165

**Figure 2:**
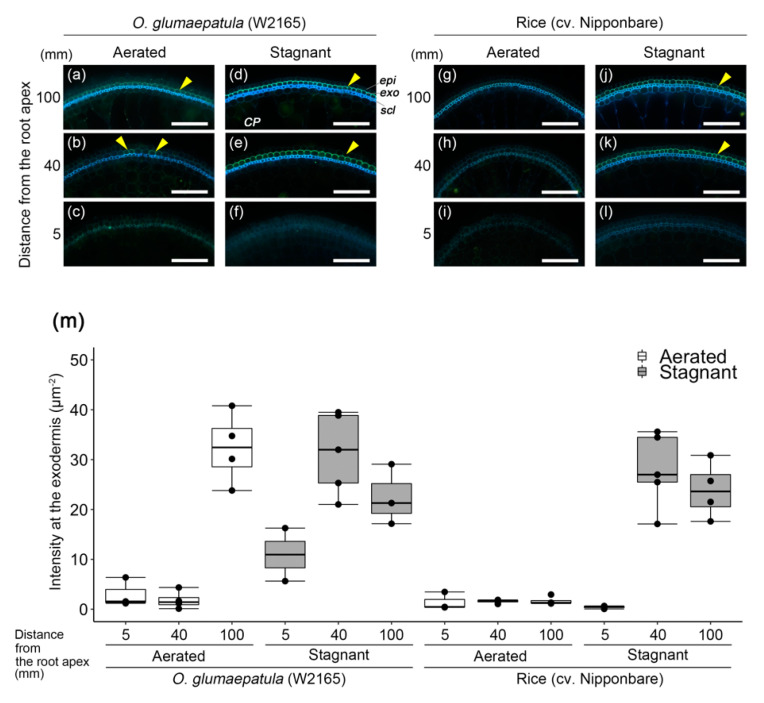
Suberization in the outer part of roots in accession W2165 of *O. glumaepatula* and rice (cv. Nipponbare) under aerated or stagnant conditions. Suberin lamellae were observed in the basal parts (95–105 mm from the root apex; (**a**,**d**,**g**,**j**), middle parts (35–45 mm from the root apex; (**b**,**e**,**h**,**k**) and root tips (2.5–7.5 mm from the root apex; (**c**,**f**,**i**,**l**) of adventitious roots of 115–120 mm length. Suberin lamellae are indicated as yellow-green fluorescence with Fluorol Yellow 088 (yellow arrowhead, mean of intensity value >20.0 μm^−2^). Blue fluorescence indicates autofluorescence. Plants were grown in aerated nutrient solution for eight days and then transferred to deoxygenated stagnant 0.1% agar solution or continued aerated solution for 13–15 days. *CP*, cortical parenchyma; *epi*, epidermis; *exo*, exodermis; *scl*, sclerenchyma. Scale bars: 100 μm. (**m**) Fluorescence intensity of Fluorol Yellow 088 at the exodermis under aerated (white box) or stagnant (grey box) conditions. Black dots indicate each raw value. n = 2–5.

The basal parts (100 mm from the root apex) of the adventitious roots of *O. glumaepatula* (W2165) were surrounded by a well-suberized exodermis, as shown by yellowish-green fluorescence of Fluorol Yellow 088 (Figure 2a). The yellow-green fluorescence at the exodermis was patchy in the middle part of the roots (40 mm from the root apex) (Figure 2b), but it was not observed at all in the root tip (5 mm from the root apex) (Figure 2c). In W2165 under stagnant conditions, both the basal and middle parts of the roots were surrounded by well-suberized exodermis (Figure 2d–e). The exodermal fluorescence intensity at 100 mm from the root apex of W2165 under aerated conditions (third box from the left in Figure 2m) was as high as the intensities in the middle and basal parts of W2165 and cv. Nipponbare (where oxygen leakage was reduced) under stagnant conditions (Figure 2m). The exodermal fluorescence intensity at 100 mm from the root apex was significantly higher in W2165 under aerated conditions than in cv. Nipponbare under aerated conditions (*p* < 0.05, *t*-test; Figure 2m).

**Figure 3:**
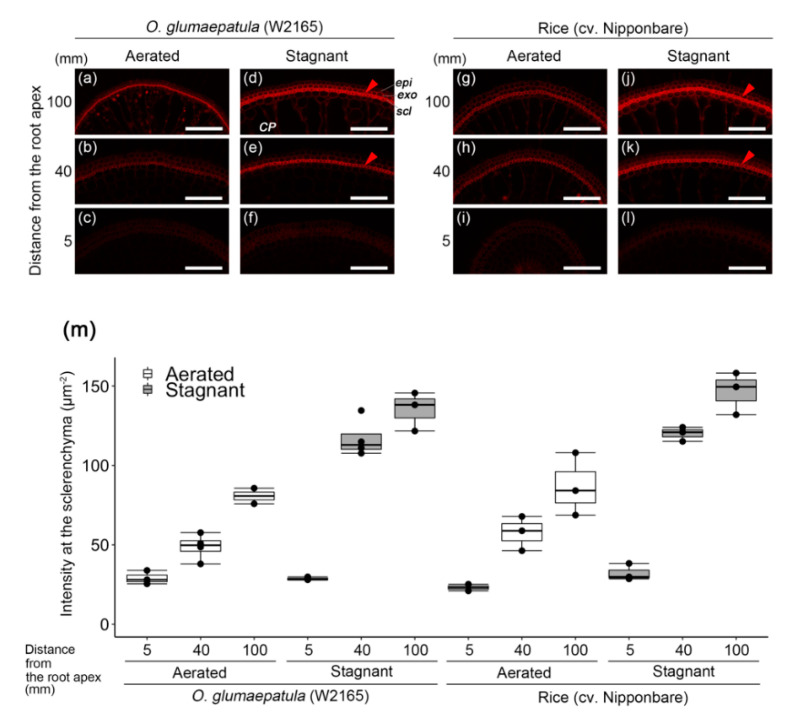
Lignification in the outer part of roots in accession W2165 of *O. glumaepatula* and rice (cv. Nipponbare) under aerated or stagnant conditions. Lignin deposits were observed in the basal parts (95–105 mm from the root apex; (**a**,**d**,**g**,**j**), middle parts (35–45 mm from the root apex; (**b**,**e**,**h**,**k**) and root tips (2.5–7.5 mm from the root apex; (**c**,**f**,**i**,**l**) of adventitious roots of 115–120 mm length. Lignin is indicated as red fluorescence with Basic Fuchsin (red arrowhead, mean of intensity value > 110.0 μm^−2^). Plants were grown in aerated nutrient solution for eight days and then transferred to deoxygenated stagnant 0.1% agar solution or continued aerated solution for 13–15 days. *CP*, cortical parenchyma; *epi*, epidermis; *exo*, exodermis; *scl*, sclerenchyma. Scale bars: 100 μm. (**m**) Fluorescence intensity of Basic Fuchsin at the sclerenchyma under aerated (white box) or stagnant (grey box) conditions. Black dots indicate each raw value. *n* = 2–4.

In W2165 under both aerated or stagnant conditions, Basic Fuchsin staining showed that lignin was more developed in the basal regions than in the more apical regions (Figure 3a–f,m) and that it was located mainly in the sclerenchyma (Figure 3a,b,d,e) and partially in the exodermis (Figure 3d). At the basal part of roots (100 mm from the root apex), the intensities of Basic Fuchsin fluorescence at the sclerenchyma under stagnant conditions were relatively higher than those under aerated conditions (Figure 3m). In cv. Nipponbare, as in W2165, lignin was well developed at the basal part of roots under stagnant conditions (Figure 3j,k). The fluorescence intensity at 100 mm from the root apex in cv. Nipponbare (9th box from the left in Figure 3m) was nearly equal in that of W2165 (third box from the left in Figure 3m). The roots of cv. Nipponbare were still leaky to oxygen at 100 mm from the root apex (Figure 1e), but the roots of W2165 were less leaky (Figure 1a). Lignification of the basal part of roots in W2165 under aerated conditions was not associated with ROL barrier formation (Figure 1a and Figure 3a).

### 2.5. Apoplastic Barrier Assay in W2165

**Figure 4:**
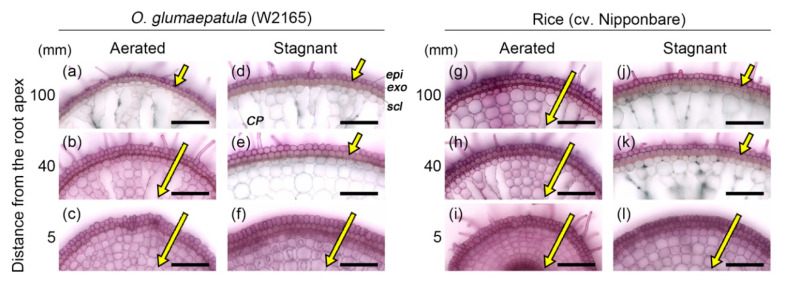
Permeability of the outer part of roots to an apoplastic tracer (periodic acid) in accession W2165 of *O. glumaepatula* and rice (cv. Nipponbare) under aerated or stagnant conditions. The permeability of the exodermis was evaluated at the basal parts (95–105 mm from the root apex; (**a**,**d**,**g**,**j**), middle parts (35–45 mm from the root apex; (**b**,**e**,**h**,**k**) and root tips (2.5–7.5 mm from the root apex; (**c**,**f**,**i**,**l**) of adventitious roots of 115–120 mm length. Purple color indicates that periodic acid penetrated into root tissues. The length of yellow arrows indicates the extent of penetration. Plants were grown in aerated nutrient solution for eight days and then transferred to deoxygenated stagnant 0.1% agar solution or continued aerated solution for 13–15 days. *CP*, cortical parenchyma; *epi*, epidermis; *exo*, exodermis; *scl*, sclerenchyma. Scale bars: 100 μm.

The ability of the ROL barrier to act as an apoplastic barrier was tested with an apoplastic tracer (periodic acid). At the basal parts of roots in W2165 under aerated and stagnant conditions, the purple color of periodic acid was detected only in the epidermal cells (Figure 4a,d,e). The penetration of the tracer was blocked at the outside of the exodermis. In cv. Nipponbare, penetration of the tracer was also blocked at the outside of the exodermis under stagnant conditions (Figure 4j,k), but not under aerated conditions (Figure 4g). Thus, the constitutive ROL barrier in W2165 also acts as an apoplastic barrier at the exodermis.

## 3. Discussion

Until this study, a constitutively formed ROL barrier had not been reported in any rice or wild rice accessions. Here, by screening four accessions of two wild rice species from wetland and non-wetland habitats, we found three accessions of *O. glumaepatula* (W2165, W2149, and W1183) that form a constitutive ROL barrier (Figure 1a–c). Because they have an AA-genome, the three accessions are good candidates for crossing with cultivated rice to identify the QTLs that regulate constitutive ROL barrier formation. Similar hybridization approaches have been used to transfer wild QTLs associated with an inducible ROL barrier formation to wheat [28,29] and maize [30], as well as to understand the genetic regulation of ROL barrier formation [30]. *Hordeum marinum*, a waterlogging-tolerant wild relative of wheat, inducibly forms an ROL barrier under stagnant conditions [31]. To obtain wheat varieties with inducible ROL barriers in the roots, wheat was hybridized with *H. marinum*, producing amphiploids [28] and disomic chromosome addition lines [29]. Under stagnant conditions, two of the amphiploids had tight ROL barriers [28], while no ROL barrier was detected in any of six disomic chromosome addition lines tested [29]. The wild maize *Zea nicaraguensis* inducibly forms an ROL barrier under stagnant conditions, but maize does not [30]. Analyses of *Z. nicaraguensis* introgression lines in the genetic background of maize identified a major locus in a segment of the short arm of chromosome 3 of *Z. nicaraguensis* as this segment conferred inducible ROL barrier formation in maize [32]. So far, little is known about the genetic regulation of constitutive ROL barrier formation. Introgression lines of another accession of *O. glumaepatula* (IRGC105668) in the genetic background of *O. sativa* (cv. Taichung 65) have also been developed to investigate other QTLs [33]. Thus, mapping ROL-related QTLs in *O. glumaepatula* may help to understand how constitutive ROL barrier formation is regulated.

Aerenchyma formation, as well as an ROL barrier formation, are key features contributing to long-distance oxygen transport and waterlogging tolerance [2,5]. Like cultivated rice, all four of the wild accessions formed aerenchyma constitutively (Table 1). Constitutive aerenchyma formation has also been observed in other wild grasses [34,35,36,37]. *Z. nicaraguensis* accessions with a higher degree of constitutive aerenchyma formation had better waterlogging tolerance than maize and the other *Z. nicaraguensis* accessions with a lower degree of constitutive aerenchyma formation [38]. Additionally, accessions of *O. glumaepatula* formed a constitutive ROL barrier (Figure 1a–c). Even in cultivated rice, forming an ROL barrier takes 24–48 h after the start of stagnant treatment [25,39]. Additionally, shorter adventitious roots (65–90 mm length roots at the commencement of treatment) need 72–120 h to complete ROL barrier formation [25]. Constitutive ROL barrier is likely to have an advantage for rapid adaptation to waterlogging or flash-flooding. Having a constitutive ROL barrier is likely to be an advantage for plants living in areas prone to flash-flooding, such as accessions of *O. glumaepatula* that grow in the floodplain of the Amazon river [40]. Thus, constitutive aerenchyma and constitutive ROL barrier of *O. glumaepatula* accessions would clearly be an advantage.

Like cv. Nipponbare (*O. sativa* vg. *japonica*), accession W1962 of *O. rufipogon* formed an inducible ROL barrier (Figure 1d). Cultivated rice (*O. sativa* vg. *japonica*) was domesticated from a specific *O. rufipogon* population in southern China thousands of years ago [41]. This raises the possibility that the inducible ROL barrier in cultivated rice is inherited from its ancestor (i.e., *O. rufipogon*). But, there are many morphological and physiological differences between *O. sativa* and *O. rufipogon* [27]. Additionally, we checked only one *O. rufipogon* accession (W1962) (Figure 1d) that came from a non-wetland habitat. *O. rufipogon* grows in deep permanent water [42]. There are over 600 accessions of *O. rufipogon* in the Oryzabase database. Thus, accessions that grow in flooding sites might be more likely to form a constitutive ROL barrier. Further investigations of ROL barrier formation in more accessions of *O. rufipogon* and other wild rice species are needed to reveal how wild species of *Oryza* acquired traits of waterlogging tolerance during the course of their evolution.

Suberin is considered a major component of ROL barriers [6]. A transcriptome analysis using laser-microdissected tissues of the outer part of roots in rice showed that many genes involved in suberin biosynthesis (but not in lignin biosynthesis) were up-regulated during ROL barrier formation in rice [43]. Moreover, a metabolomic analysis in rice clearly showed that rice roots that form an ROL barrier accumulate malic acid and very long-chain fatty acids, which are substrates for suberin biosynthesis [44]. In the present study, the constitutive ROL barrier of W2165 was closely associated with suberization (Figure 2) but not associated with lignification (Figure 3). This is in agreement with observations that suberized exodermis was associated with constitutive ROL barrier formation in wild *Echinochloa* accessions [13] and a tropical forage grass (*Urochloa humidicola* [45]).

Moreover, suberin lamellae inhibited the infiltration of an apoplastic tracer (periodic acid) (Figure 4), suggesting that the ROL barrier can prevent the entry of phytotoxic compounds from the soil. Similar findings were reported for the constitutive ROL barrier in a wild *Echinochloa* species [13] and inducible ROL barrier in rice [46] and *Z. nicaraguensis* [32]. The constitutive ROL barrier of W2165 would have the advantage of detoxifying and preventing the infiltration of toxic ion. The present results open the door to further QTL studies to identify the genes involved in constitutive ROL barrier formation and thus the development of more flood-tolerant varieties of rice.

## 4. Materials and Methods

### 4.1. Plant Materials

The *Oryza glumaepatula* accessions (W2165, W2149, and W1183) and *O. rufipogon* accession (W1962) used in this study were kindly provided by the National Institute of Genetics, Japan. For a control that forms an inducible ROL barrier, we used rice (*O. sativa* L. cv. Nipponbare).

### 4.2. Growth Conditions

To break dormancy, *O. glumaepatula* and *O. rufipogon* seeds were incubated at 35 °C for five days and rice cv. Nipponbare seeds were incubated at 50 °C for three days. Seeds were sterilized for 30 min in 0.6% (*w*/*v*) sodium hypochlorite, washed thoroughly with deionized water, and for imbibition, placed in Petri dishes (8.5 cm diameter) containing about 6 mL of deionized water (about 1 mm water-depth) at 28 °C under darkness. The plants were grown in a controlled-environment chamber under constant light to avoid effects of circadian rhythm on gene expression (24 h light, 28 °C, relative humidity over 50%, photosynthetic photon flux density at 248.8 μmol m^−2^ s^−1^). One day after imbibition, seeds were placed on a stainless mesh with attached floats floating on an aerated quarter-strength nutrient solution [24,25] and exposed to light. For the next phase, a soft sponge was floated on a container (380 mm × 260 mm × 160 mm high) containing aerated full-strength nutrient solution. Vertical slits were cut into the edges of the sponge. Six days after imbibition, each plant was inserted into the slit so that the roots were submerged, and the shoot protruded through the sponge into the light. To evaluate constitutive ROL barrier formation under aerated conditions, eight days after imbibition, plants were transplanted into aerated nutrient solution in 5-L pots (120 mm × 180 mm × 250 mm high, three plants per pot) for an additional 13–15 days. In each pot, a rectangular 2 cm-thick piece of foam was placed on the solution, and aluminum foil was placed on the top of the foam to keep the solution dark. Vertical cuts were made on three sides of the foam to accommodate the stems. Then three plants were transferred to each pot, sliding the stems into the cuts. In this way, the roots were kept dark. The nutrient solution was renewed every seven days.

To evaluate inducible ROL barrier formation under stagnant conditions, eight days after imbibition, plants were transplanted into stagnant deoxygenated nutrient solution in 5 L pots for 13–15 days. The stagnant solution was prepared by adding 0.1% (*w*/*v*) agar (not enough to cause solidification) to the nutrient solution and boiling the solution to dissolve the agar. The low agar concentration produced a viscous liquid rather than a gel. By preventing convective movements, the solution mimics the changes in gas composition found in waterlogged soils (i.e., decreased oxygen, increased ethylene) [47]. The solution was poured into the pots and deoxygenated by bubbling N_2_ gas gently through two air stones at a flow rate of about 2.2 L min^−1^ for 15 min per pot. The dissolved oxygen (DO) level was confirmed to be less than 1.0 mg L^−1^ by DO meter (SG6-ELK, Mettler Toledo, Greifensee, Switzerland).

### 4.3. ROL Barrier Formation

Radial oxygen loss from adventitious roots was measured with Pt cylindrical root-sleeving O_2_ electrodes [48,49]. The roots were relatively young (115 to 120 mm long) and had few or no lateral roots. The plants were placed in clear plastic boxes (55 mm × 55 mm × 300 mm high) fitted with rubber lids. The boxes were filled with an O_2_-free medium containing 0.1% (*w*/*v*) agar, 0.5 mM CaSO_4_, and 5 mM KCl. The plant was inserted through the hole, fixing the shoot base to the rubber lid. Thus, the shoot was in the air, and the root was in the medium. An adventitious root was inserted through the cylindrical root-sleeving O_2_ electrode (internal diameter, 2.25 mm; height, 5.0 mm). Root diameters at the point of oxygen measurement were measured with a micrometer caliper. Following Fick’s law, the rate of ROL was calculated with the following equation [48]:

$$ROL=-\frac{4.974I}{A},$$

where *ROL* is radial oxygen loss (O_2_ ng cm^−2^ root surface min^−1^), *I* is the diffusion current (µA) with the root in the electrode minus the diffusion current (µA) without the root (current of background), and *A* is the surface area of the root within the electrode (cm^2^). The oxygen electrode was 5 mm long, and it was placed at 5, 10 or 20 mm intervals along the root. So, for example, when the electrode was centered at 10 mm, it measured oxygen loss from 7.5 to 12.5 mm. The experiment was conducted in a lighted room kept at a constant 23 °C.

### 4.4. Aerenchyma Formation

The roots used for aerenchyma measurements were the same ones that were previously used for ROL measurements with the O_2_ electrode. Root segments at the basal parts (95–105 mm from the root apex) were prepared from the adventitious roots. Root cross-sections were prepared by hand sectioning with a razor blade. Root cross-sections were photographed using a microscope (Axio Imager.A2, Carl Zeiss, Oberkochen, Germany) under white light with a CCD (charge-coupled device) camera (AxioCam MRc CCD, Carl Zeiss). Areas of cortex and aerenchyma in the cross-section were measured using Fiji (version 2.0.0-rc-69/1.52p), and the percentage of the cortex occupied by aerenchyma was calculated from the cross-sectional areas.

### 4.5. Histochemical Staining

The adventitious roots (115–120 mm length) in which ROL was measured with the O_2_ electrode were cut at the root-shoot junction. Their basal parts (95–105 mm from the root apex), middle parts (35–45 mm from the root apex), and root tips (2.5–7.5 mm from the root apex) were each embedded in 5% (*w*/*v*) agar, respectively. Root cross-sections of 100 μm thickness were made using a vibrating microtome (Leica VT1200S, Leica Biosystems, Wetzlar, Germany). The cross-sections were made transparent by incubating them in lactic acid saturated with chloral hydrate at 70 °C for 60 min [50]. To detect suberin lamellae, we used 0.01% (*w*/*v*) Fluorol Yellow 088 in polyethylene glycol 400 as described previously [51]. Suberin lamellae were visualized as a yellowish-green fluorescence excited by ultraviolet (UV) light. For quantification, all cross-sections were photographed with a fluorescence microscope with the following settings [Exposure time: 0.8438 sec; an 02 UV filter set (Excitation G 365 nm, Beamsplitter FT 395, Emission LP 420), an Axio Imager.A2 and an AxioCam MRc CCD camera (all Carl Zeiss)]. Lignin was stained with Basic Fuchsin [52], which causes it to fluoresce red. Before lignin staining, cross-sections were made transparent by incubating them overnight in ClearSee solution at room temperature [53]. Cross-sections were incubated overnight in 0.2% (*w*/*v*) Basic Fuchsin (Sigma-Aldrich) dissolved in ClearSee solution at room temperature as described previously [54]. Before the observation, the cross-sections were gently washed at least five times with ClearSee solution. Lignin was imaged on a confocal microscope (LSM510 META, Carl Zeiss; Excitation: 543 nm, Detection: 565–651 nm).

### 4.6. Quantification of Fluorescence Intensity

All images were obtained with the same section thickness, same exposure time, and the same laser power for excitation. In the images obtained by the fluorescence and confocal microscopes (both Carl Zeiss), the fluorescence intensities at each pixel were recorded as 12 bit and 16 bit values, respectively (Supplemental Figures S1a and S2a, respectively). Fluorescence intensities were quantified using Fiji.

To quantify Fluorol Yellow 088 fluorescence (Supplemental Figure S1a), impulse noise was removed from each image with a median filter (Fiji command: Median, radius: 0.5 pixels). Then, the 12 bit color root images were split into red, green, and blue images by Fiji (Fiji command: Split Channels) (Supplemental Figure S1b). To quantify yellow fluorescence, the sum of the red and green intensities at each pixel was subtracted from the blue intensity (Fiji command: Image Calculator). The images were then converted to 8 bit values (0–255) (Fiji command: 8 bit) (Supplemental Figure S1c). The background intensities (i.e., area of the solvent without root cross-section as a red rectangle in Supplemental Figure S1c) in the images were measured (Fiji Command: Measure), and the mean value was 14.733 ± 1.869 (mean ± standard deviation). Background noise was removed by subtracting the mean intensity of blank images (Fiji command: Subtract, value: 14.733) (Supplemental Figure S1d).

To quantify Basic Fuchsin fluorescence (Supplemental Figure S2a), impulse noise was removed from each image with a median filter (Fiji command: Median, radius: 0.5 pixels). Then, intensities of the 16 bit monochrome images were converted to 8 bit values (Fiji command: 8 bit) (Supplemental Figure S2b). The background intensities (i.e., area of the solvent without root cross-section as a red rectangle in Supplemental Figure S2b) in the images were measured (Fiji Command: Measure), and the mean value was 10.934 ± 2.822 (mean ± standard deviation). Background noise was removed by subtracting the mean intensity of blank images (Fiji command: Subtract, value: 10.934).

Fluorescence intensities were calculated for regions of interest (ROIs) that consisted of 20 selected cells. Fluorescence intensity (μm^−2^) was calculated as *F*/*A_ROI_*, where *F* is the dimensionless sum of fluorescence intensities at each pixel in the ROI, and *A_ROI_* is the area of the ROI (μm^2^). For Fluorol Yellow 088, the ROI was in the exodermis (Supplemental Figure S1d) and for Basic Fuchsin, the ROI was in the sclerenchyma (Supplemental Figure S2c).

### 4.7. Permeability Test

Adventitious roots (115–120 mm length) were cut at the root–shoot junction. The permeabilities of the exodermal layers at the basal parts (95–105 mm from the root apex), middle parts (35–45 mm from the root apex), and root tips (2.5–7.5 mm from the root apex) were assessed with an apoplastic tracer, periodic acid, as described previously [13]. Periodic acid that penetrated the root tissue was visualized as a purple color under white light with the above microscope and camera.

### 4.8. Statistical Analysis

Means of aerenchyma formation (% aerenchyma/cortex) were compared with one-way analysis of variance (ANOVA) and Tukey’s honest significant difference (HSD) for multiple comparisons at the 5% probability level or with a two-sample *t*-test at the 5% probability level. Means of fluorescence intensities of Fluorol Yellow 088 or Basic Fuchsin between accession W2165 of *O. glumaepatula* and cv. Nipponbare was compared with a two-sample *t*-test at the 5% probability level. The data were analyzed with R version 3.5.1 [55].

## Figures and Tables

**Table 1:** Aerenchyma formation (% aerenchyma/cortex) in accessions of *O. glumaepatula*, *O. rufipogon*, and rice (cv. Nipponbare) under aerated or stagnant conditions.

Species	Accession	Aerenchyma Formation (% aerenchyma/cotex)		
		Aerated	Stagnant	*t*-Test
*O. glumaepatula*	W2165	48 ± 3 ^c^	46 ± 5 ^A^	n.s.
	W2149	32 ± 5 ^a*,*b^	52 ± 4 ^A^	*
	W1183	21 ± 1 ^a^	49 ± 6 ^A^	*
*O. rufipogon*	W1962	38 ± 2 ^b*,*c^	52 ± 2 ^A^	*
*O. sativa*	Nipponbare	48 ± 2 ^c^	64 ± 4 ^A^	*

Aerenchyma formation (% aerenchyma/cortex) at 100 mm from apex of 115–120 mm-length roots. Asterisks denote significant differences between means of aerated and stagnant conditions (two-sample *t*-test, *: *p* < 0.05). n.s.: not significant. Different lower-case and upper-case letters denote significant differences among accessions under aerated and stagnant conditions, respectively (*p* < 0.05, one-way analysis of variance (ANOVA) and Tukey’s honest significant difference (HSD) for multiple comparisons). Plants were grown in aerated nutrient solution for eight days and then transferred to deoxygenated stagnant 0.1% agar solution or continued aerated solution for 13–15 days. Mean ± SE. *n* = 3 or 4.

## Supplementary Materials

The following are available online at https://www.mdpi.com/2223-7747/9/7/880/s1, Figure S1: Procedure for quantifying Fluorol Yellow 088 fluorescence intensity; Figure S2: Procedure for quantifying Basic Fuchsin fluorescence intensity.
