# Supplementary material for: Some Accessions of Amazonian Wild Rice (Oryza glumaepatula) Constitutively Form a Barrier to Radial Oxygen Loss along Adventitious Roots under Aerated Conditions
Source: Plants (Basel). 2020 Jul 13;9(7):880. doi: 10.3390/plants9070880 (PMC7412225; doi:10.3390/plants9070880)
Supplement: Supplementary file 1 [file plants-09-00880-s001.pdf]

Supplementary Materials

Some Accessions of Amazonian Wild Rice (*Oryza glumaepatula*) Constitutively Form a Barrier to Radial Oxygen Loss along Adventitious Roots under Aerated Conditions

Masato Ejiri, Yuto Sawazaki and Katsuhiro Shiono * *

*Corresponding author: Katsuhiro Shiono (E-mail: shionok@fpu.ac.jp)

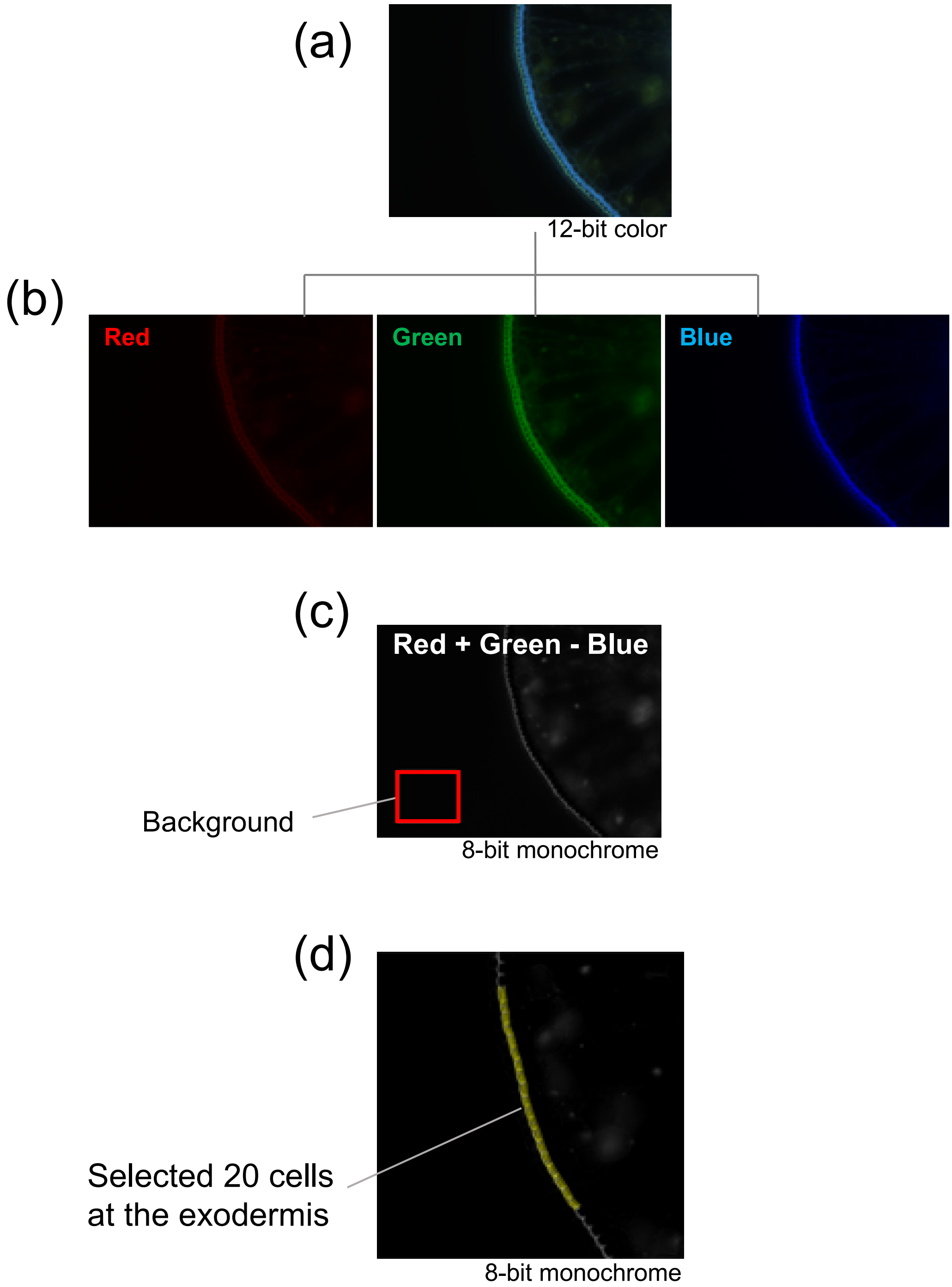

Supplemental Figure 1. Procedure for quantifying Fluorol Yellow 088 fluorescence intensity. **(a)** Original 12-bit color image. **(b)** Splitting the image into red, green, and blue. **(c)** Calculation of yellow intensity. For each pixel, the yellow intensity was calculated as the sum of the red and green intensities minus the blue intensity. **(d)** Image to select 20 cells at the exodermis as a region-of-interest (ROI).

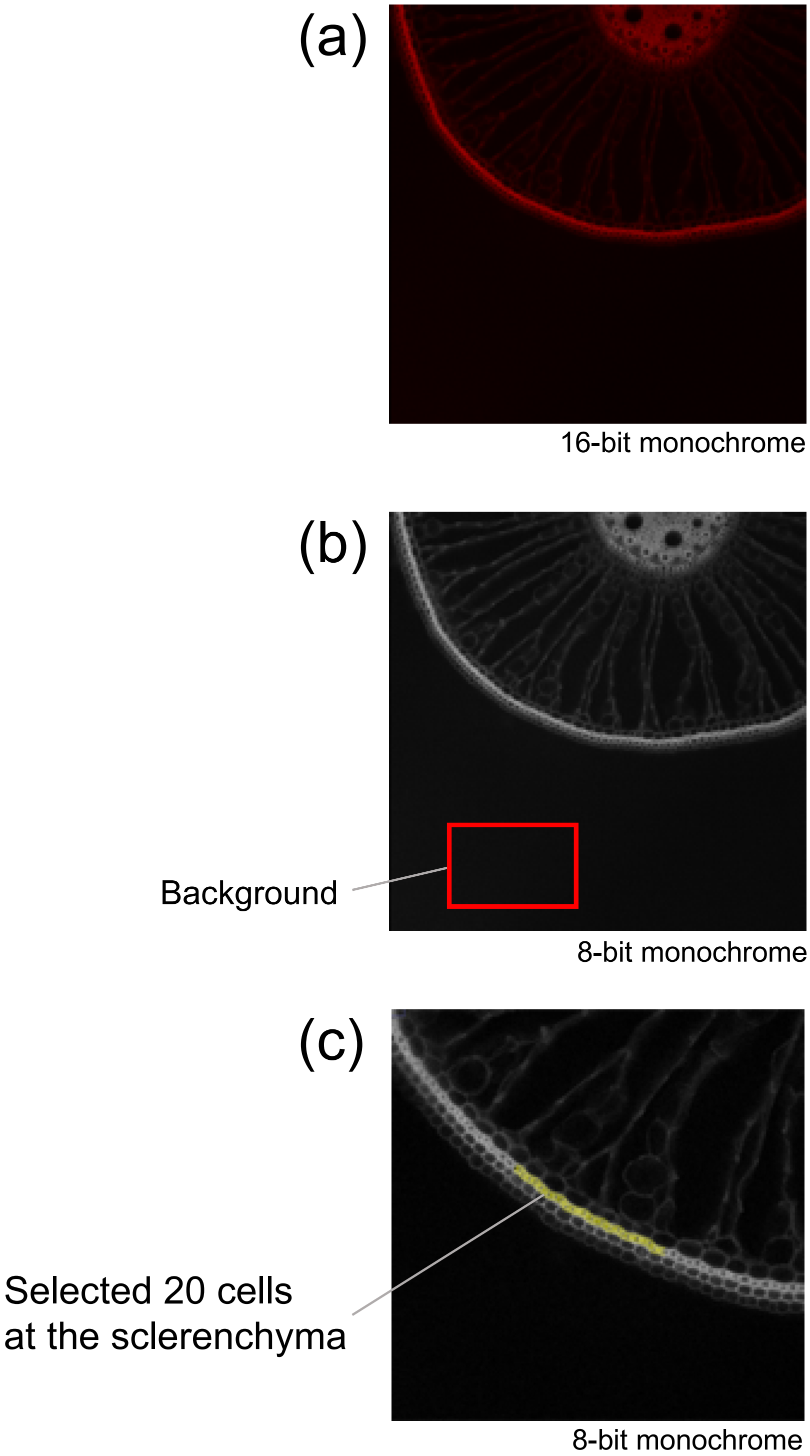

Supplemental Figure 2. Procedure for quantifying Basic Fuchsin fluorescence intensity. **(a)** Original 16-bit monochrome image as shown by red pseudo-color. **(b)** 8-bit monochrome image. **(d)** Image to select 20 cells at the sclerenchyma as a region-of-interest (ROI).
